# Short-term efficacy of physical interventions in osteoarthritic knee pain. A systematic review and meta-analysis of randomised placebo-controlled trials

**DOI:** 10.1186/1471-2474-8-51

**Published:** 2007-06-22

**Authors:** Jan M Bjordal, Mark I Johnson, Rodrigo AB Lopes-Martins, Bård Bogen, Roberta Chow, Anne E Ljunggren

**Affiliations:** 1 Faculty of Health and Social Sciences, Institute of Physiotherapy, Bergen University College, Moellendalsvn. 6, 5009 Bergen Norway; 2 Department of Public Health and Primary Health Care, Section of Physiotherapy Science University of Bergen, Kalfarveien 31, 5018 Bergen, Norway; 3 Faculty of Health, Leeds Metropolitan University, Civic Quarter, Leeds, LS1 3HE, UK; 4 Department of Pharmacology, Institute of Biomedical Sciences, University of São Paulo. Av. Prof. Lineu Prestes, 1524, Butantan, 05508-900São Paulo – SP, Brazil; 5 Haraldsplass Deaconal Hospital, Physiotherapy Unit, Ulriksdal 10, 5009 Bergen, Norway; 6 Castle Hill Medical Center, 103 Malton Rd, Beecroft, New South Wales, 2119 Australia

## Abstract

**Background:**

Treatment efficacy of physical agents in osteoarthritis of the knee (OAK) pain has been largely unknown, and this systematic review was aimed at assessing their short-term efficacies for pain relief.

**Methods:**

Systematic review with meta-analysis of efficacy within 1–4 weeks and at follow up at 1–12 weeks after the end of treament.

**Results:**

36 randomised placebo-controlled trials (RCTs) were identified with 2434 patients where 1391 patients received active treatment. 33 trials satisfied three or more out of five methodological criteria (Jadad scale). The patient sample had a mean age of 65.1 years and mean baseline pain of 62.9 mm on a 100 mm visual analogue scale (VAS). Within 4 weeks of the commencement of treatment manual acupuncture, static magnets and ultrasound therapies did not offer statistically significant short-term pain relief over placebo. Pulsed electromagnetic fields offered a small reduction in pain of 6.9 mm [95% CI: 2.2 to 11.6] (n = 487). Transcutaneous electrical nerve stimulation (TENS, including interferential currents), electro-acupuncture (EA) and low level laser therapy (LLLT) offered clinically relevant pain relieving effects of 18.8 mm [95% CI: 9.6 to 28.1] (n = 414), 21.9 mm [95% CI: 17.3 to 26.5] (n = 73) and 17.7 mm [95% CI: 8.1 to 27.3] (n = 343) on VAS respectively versus placebo control. In a subgroup analysis of trials with assumed optimal doses, short-term efficacy increased to 22.2 mm [95% CI: 18.1 to 26.3] for TENS, and 24.2 mm [95% CI: 17.3 to 31.3] for LLLT on VAS. Follow-up data up to 12 weeks were sparse, but positive effects seemed to persist for at least 4 weeks after the course of LLLT, EA and TENS treatment was stopped.

**Conclusion:**

TENS, EA and LLLT administered with optimal doses in an intensive 2–4 week treatment regimen, seem to offer clinically relevant short-term pain relief for OAK.

## Background

Osteoarthritis of the knee (OAK) is the most common type of osteoarthritis (OA)[1], and its prevalence is rising in parallel with the increasing age of the population [2]. The condition is associated with pain and inflammation of the joint capsule [3], impaired muscular stabilisation [4,5], reduced range of motion [6], and functional disability.

European League Against Rheumatism (EULAR) recommendations state that both pharmacological and non-pharmacologial interventions are needed for optimal treatment of OAK with at least 33 potentially effective interventions at the clinicians' disposal [7]. Ten of these interventions are listed as non-pharmacological and 5 of these non-pharmacological interventions are physical agents: acupuncture; low level laser therapy (LLLT); pulsed electromagnetic fields (PEMF, including shortwave therapy SWT); transcutaneous electrical nerve stimulation (TENS), and ultrasound (US). While paracetamol, opioids and coxibs receive recommendations based on the second highest level of evidence (1B), no physical agents are recommended in spite of being listed as having the same evidence level (1B).

Inadequate dosageand inappropriate procedural technique can contaminate the findings of RCTs of physical agents but the EULAR analysis did not account for this. Recent findings suggest that most physical agents exhibit fairly distinct dose-response patterns, and failure to account for adequacy of TENS [8] and LLLT [9] interventions can markedly reduce ES estimates. Indeed, evidence-based guidelines for dosage and treatment procedures and the conduct of systematic reviews have been published for LLLT [10], and for acupuncture [11].

An appropriate approach would then be to investigate the short-term efficacy of physical agents for OAK, for all trials with each intervention and then to make sub-group analyses for trials according to their compliance with adequate dosageand procedural recommendations. Consistency in trial design and in the selection and timing of outcome measures must be assured to allow for comparison between interventions [12]. The selected meta-analysis methodology was identical to that previously used by our group to assess common pharmacological interventions for OAK [13].

## Methods

### Review protocol specification

A detailed review protocol was specified prior to analysis. This included a sequential three-step reviewing procedure of 1) harvesting randomised placebo-controlled trials where patients were treated with specified interventions for knee ostoarthritis, 2) evaluating their methodological quality according to predefined criteria, and 3) calculating their pooled effect as the weighted mean difference (WMD) in change between intervention and placebo in mm on a 100 mm visual analogue scale (VAS).

### Literature search

A specified literature search was performed from 1966 through April 2006 on Medline, Embase, Cochrane Controlled Trials Register for RCTs, CINAHL, Database of Abstracts of Reviews of Effectiveness (DARE), International Network of Agencies for Health Technology Assessment (INAHTA) database, The Physiotherapy Evidence Database (PEDro), National Guideline Clearinghouse (NGC), PRODIGY Guidance, and NICE (National Institute for Clinical Excellence). In addition, hand searches were performed in the journal Laser Therapy from 1994, and in books of abstracts from congresses arranged after 1990 by the World Confederation of Physical Therapy and World Association for Laser Therapy.

The following search string was used: Osteoarthritis OR osteoarthrosis OR knee OR exercise OR electrotherapy OR laser therapy OR light therapy OR ultrasound OR electrostimulation OR transcutaneous electrical nerve stimulation OR electromagnetic AND randomized OR randomised.

In addition, handsearches of national Scandinavian physiotherapy journals, conference abstracts and reference lists of systematic reviews were performed, and experts in the field were consulted. No language restrictions were applied with papers in English, German and Scandinavian languages eligible for inclusion.

### Inclusion criteria

The trials were subjected to 6 inclusion criteria:

#### 1. Diagnosis

A statement in the report that knee osteoarthritis had been verified by clinical examination according to the American College of Rhematology criteria and/or by x-ray.

#### 2. Symptom duration

More than 3 months.

#### 3. Trial design

Randomised blinded placebo-controlled parallel and cross-over groups design.

#### 4. Outcome measures

Primary outcome measure: Pain intensity within 4 weeks of treatment start scored on the Western Ontario and McMaster Universities osteoarthritis index (WOMAC) subscale of pain, or on a 100 mm VAS for global or walking pain.

Secondary outcome measure: Pain intensity, as measured for the primary outcome measure, at 5–12 weeks follow-up.

#### 5. Threshold levels for clinical relevance

Mean threshold for OAK patients reporting "minimal clinical important improvement" has been determined to 19.9 mm on VAS [14]. Likewise, the mean threshold for inducing a categorical change from "no change" to "slight improvement" has been determined to be 12.7 mm [15], while the mean threshold for "minimal perceptible clinical improvement" is determined to be 9.7 mm [16].

#### 6. Intervention groups, including criteria for modality-specific optimal dosage

##### Acupuncture

Interventions which produced somatic stimulation of 'acupuncture points' were included; i.e. manual or electrical dry needling.

Criteria for optimal dose (i.e. compliance with adequate dosageand procedural recommendations) were: manual or electrical dry needling of acupuncture 3 or more acupuncture points as defined in Traditional Chinese Medicine and performed by an acupuncturist with at least 2 years clinical experience. As it is plausible that manual acupuncture and electro-acupuncture trigger different biological mechanisms, we decided to group and assess manual acupuncture and electro-acupuncture separately. Categorisation as electro-acupuncture demanded electrical current intensity to be at a strong, near noxious level, which has been shown to be more effective than a low intensity level [17].

##### Low level laser therapy (LLLT)

Criteria for optimal dose: GaAs 904 nm infrared pulse lasers = intensities between 12–60 mW/cm^2 ^and doses between 1 – 4 Joule per session; GaAlAs 780–860 nm infrared pulse lasers = intensities between 30–200 mW/cm^2 ^and doses between 6 – 24 Joule per session.

These doses are based on optimal location-specific dose ranges for osteoarthritis when the joint capsule is exposed [9] and dosage recommendations from World Association of Laser Therapy for pain relief[18].

##### Pulsed electromagnetic fields (PEMF), including shortwave therapy (SWT)

###### SWT (27 MHz)

Criteria for optimal dose: intensity between 14.2–76.7 Watts, pulse frequency between 100–800 Hz, treatment time 20–30 minutes and doses between 17–138 kJoule per session. These doses are based on a review of clinical trial literature to determine optimal treatment procedures and dose ranges for shortwave (27 MHz)[19].

##### PEMF other than SWT

Criteria for optimal dose: There is a lack of consensus of optimal doses for intensity, so PEMF (other than SWT) delivered at any intensity was included. Frequencies between 10 and 200 Hz in line with those used in most animal studies.

##### Electrical stimulation using surface electrodes (TENS)

Interventions which delivered electrical currents in the milliampere range across the intact surface of the skin to stimulate nerves innervating the knee joint (L4-5, S1; [20]) were included providing a standard TENS device or an interferential current stimulator was used [21]. Interventions using any other TENS-like device were excluded because of the absence of a plausible physiological rationale (e.g. microcurrent electrical stimulation, high voltage pulse (galvanic) currents, high voltage TENS pens, transcranial electrical stimulation, transcutaneous spinal electroanalgesia (TSE), H-wave therapy and action potential simulation [21]). No restrictions were placed on the electrode types.

Criteria for optimal dose: a strong, near-noxious intensity, pulse frequencies between 1–150 Hz, treatment time at least 20 minutes per session in at least 5 sessions. These doses are based on a meta-analysis with sub group analysis for optimal dose for TENS [8].

##### Ultrasound therapy

Interventions which delivered mechanical vibration using an ultrasound device at frequencies between 1.0–3.0 MHz

Criteria for optimal dose: intensity 0.1–3 W/cm2, continuous or pulsed output, treatment time between 3–20 minutes and doses between 18–540 Joules per session. These doses are based on those commonly reported in clinical literature as optimal dose range has yet to be established [22,23].

##### Static magnets

Criteria for optimal dose for this modality remain uncertain, as does their anatomic location for placement on the human body.

##### Placebo control groups

Reports that stated that they had included a placebo or sham control were included. For LLLT, PEMF, TENS and US reports were checked to ensure that the placebo/sham intervention was inert in the form of an identical device delivering no output (i.e. a dummy device). For acupuncture, sham interventions were considered inert if they used non-acupuncture points and superficial needling (≤ 2 mm) or a specifically designed placebo needle. For sham magnet therapy, identical-looking devices without any or insignificant magnetic fields were considered.

##### Assessment of methodological quality

A criteria-list of methodological criteria was used for assessment of trial quality [24]. Assessments of trial methodology were made by two independent reviewers (JMB and RABL-M). No specific cut-off limit for method scores was pre-planned as criterion for exclusion.

#### Outcome measure extraction

The change in overall pain intensity between the active intervention group and placebo was used. If more than one attainable outcome measurement was obtained in the first 4 weeks after treatment started, the time point corresponding to the largest effect values was selected. If data on overall pain intensity were missing, data were obtained as a mean of the 5 items on the WOMAC pain subscale. If WOMAC data were registered on non-continuous (categorical, Likert) scales, they were converted to 100 mm VAS and checked against other subscales and overall WOMAC score, as this has been found to have good internal consistency [25]. If overall pain or WOMAC pain subscale data were unavailable, pain on movement was used as registered on a 100 mm VAS.

##### Statistical analysis of pain-relieving effect

Mean differences of change for intervention groups and placebo groups and their respective standard deviations (SD) were included in a statistical pooling. If variance data were not reported as SDs, they were re-calculated algebraically from the trial data of sample size and other variance data such as p-values, t-values, standard error of mean, or 95% confidence intervals [CI]. As a control measure for the stability of the small (n < 40) trials results, we substituted reported SDs (or other variance data) with the arithmetic mean SD from the other trials with the same intervention if SD was lower than the the arithmetic mean [26].

Results were presented as weighted mean difference (WMD) between intervention and placebo with 95% CI in mm on VAS, i.e., as a pooled estimate of the mean difference in change between the treatment and the placebo groups, weighted by the inverse of the variance for each study (Fleiss 1993). A fixed effects model was applied.

##### Subgroup analysis

In order to give as precise effect estimates as possible, care was taken to investigate discrepancies in trial samples and interventions. The validity of heterogeneity tests is equivocal, and their results were only used to support subgrouping in cases where clinical and methodological quality heterogeneity was evident. Heterogeneity was tested using Q-values, and statistical significance was defined at the 0.05 level for each intervention. To analyse heterogeneity and effect size for each intervention, trials were then subgrouped according to baseline pain, methodological quality, adequate dosageand procedural recommendations for each physical agent using the criteria listed previously (see criteria for optimal dose). Subgroup analyses were also performed for results during the 5–12 week follow-up period, and for funding sources.

##### Publication bias analysis

Effect size plots were used as a graphical test in order to detect possible publication bias [27,28].

#### Outcome measures

1) Best reduction in pain intensity during the first 4 weeks after initiation of treatment scored on the subscale of pain on the Western Ontario and McMaster Universities osteoarthritis index (WOMAC) [29] or on a 100 mm visual analogue scale (VAS) for one, or the mean score of two or more pain dimensions. Variance was calculated from the trial data and given as 95% confidence intervals [95% CI] in mm on VAS. Effect size within 4 weeks was defined as a pooled estimate of the difference in change between the mean of the treatment and the placebo control groups, weighted by the inverse of the standard deviation for each study, i.e. weighted mean difference of change between groups.

2) Follow-up results at 1–12 weeks after end of treatment were used for pain intensity (as described under 1) or categorical data of global health status. Improved global health status was defined as any one of the following categories: "improved", "good", "better", "much improved", "pain-free","excellent". The numbers of "improved" patients were then pooled to calculate the relative risk for change in health status. A statistical software package (Revman 4.2) was used for calculations.

## Results

### Included studies

**Figure 1:**
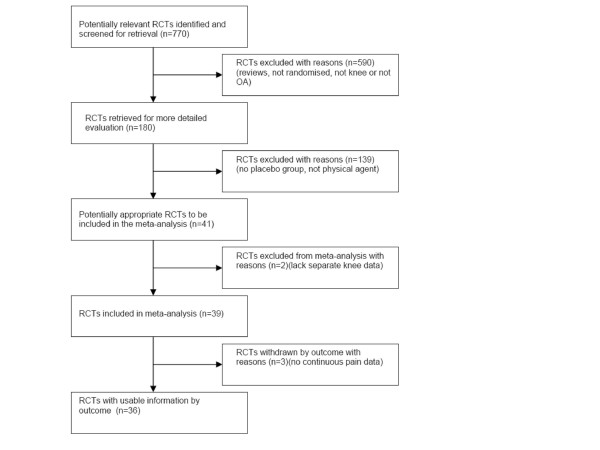
**Quorum flow chart**. Quorum flow chart showing the selection process of the review.

The literature search identified 770 potentially relevant articles that were assessed by their abstracts. 590 abstracts were excluded as irrelevant, and 180 full trial reports were evaluated. 41 trials met our inclusion criteria. However, five trials were subsequently excluded: One TENS-trial with a positive result was excluded for not giving more than a single treatment [30]. One LLLT trial with a negative result [31] and one trial with a positive result [32], were excluded for not registering continuous pain data and not giving separate data on knee osteoarthritis respectively. One SWT trial was excluded for not presenting OAK data separately [33], while another did not present pain data [34] (Figure 1

Thirty-six randomised controlled trials satisfied all our criteria for inclusion. Eleven trials were performed with TENS (n = 425), 8 trials were performed with LLLT (n = 343), 4 trials were performed with manual acupuncture (MA). One of these 4 MA trials had been recategorised because it used a weak electrical current at a comfortable level (personal communication dr. Lao). Three trials met our criteria for electro-acupuncture (EA). One trial was performed with ultrasound therapy (n = 74), 7 trials were performed with PEMF (n = 487) and two trials (n = 162) were performed with static magnets (MA). A list of included trials and their demographic data and their treatment characteristics is summarised in Table 1.

**Table 1:** Study characteristics and the distribution of trials for each intervention providing data within 4 weeks, included patients on active treatment, Q-values from heterogeneity tests, mean methodological scores, mean age of patients and baseline pain on a 100 mm visual analogue scale (VAS). (*) One trial with electroacupuncture used too low electrical stimulation intensity according to optimal treatment criteria (Berman et al. 2004), and consequently was classified as manual acupuncture.

Type of intervention	Total number of trials	Total number of patients	Number of trials with optimal treatment	Number of patients receiving optimal treatment	Mean methodological quality [range] (max score 5)	Mean age (years)	Q-values and p-values in hetero-geneity tests	Mean baseline pain on 100 mm VAS †
TENS including IF	11	425	7	277	3.3 [1–5]	63.6	60.8 (p < 0.001)	63.8
Electro-acupuncture	3	242	3	242	3.6 [3–5]	62.9	1.1 (p = 0.58)	62.7
Manual acupuncture	4	691	4	691	3.9 [3–5]	66.1	4.5 (p = 0.34)	54.7
Low Level Laser therapy	8	343	5	222	3.5 [2–5]	66.9	36.4 (p < 0.001)	66.7
Pulsed electromagnetic fields	7	487	7	487	4.4 [3–5]	64.2	9 (p = 0.18)	63.3
Ultrasound	1	74	1	74	4	67.5	n.a.	53.0
Static magnets	2	172	2	172	4 [4]	65.6	1.9 (p = 0.22)	59.7
Total and means	36	2434	24	2165	3.8	65.1		62.9†

* = Mean † = Weighted mean

### Quality of extracted data

One small trial with MA and one small trial with TENS reported unreasonably low SD values. For this reason, their SDs were substituted with the arbitrary mean SD for their intervention type [35,36]. All included trials reported variance data which allowed for algebraic calculation of SDs. One trial reported median values rather than mean values [37].

### Patient sample demographics and baseline status

The median of the reported mean age of patients was 65.1 years and 69.9% were female. Patients with grades 2–4 of roentgenological OA severity [38] were included. Eight included trials did not report baseline pain. The mean baseline pain scores on VAS were highest for LLLT (66.7 mm) and lowest for ultrasound therapy (53.0 mm) (Table 1).

### Methodological quality

The mean methodological quality scores across all included trials was 3.8 [range 1–5] out of a maximum score of 5 on the Jadad scale. TENS had the lowest mean score of 3.3 mainly due to the scores of 1 and 2 in two cross-over trials with blinding problems [36,39]. PEMF trials scored the highest mean value of 4.4. The most frequent shortcomings in the included trials were: omitting a description of the randomisation procedure, a lack of concealed allocation to groups and/or inadequate blinding. The results of the combined methodological quality score is summarised for each therapy is summarised in Table 1, and for each individual trial in Tables 2 to 8.

**Table 2:** Characteristics of trials of TENS for pain relief in patients with knee osteoarthritis

**First author, publication year**	**Type**	**No of patients on active therapy (n = 259)**	**Method quality**	**Treat-ment period (weeks)**	**Mean baseline pain (mm VAS)**	**Best mean difference (95% CI) of change over placebo (mm VAS)**	**Outcome time points (in weeks, max. effect in bold)**
Adedoyin-03	IF	15	3	4	81.0	25.4 (8.2 to 42.6)	1, 2, 3,**4**
Cheing-02	TENS	16	4	2	-	8.4 (0.7 to 16.1)	**2**, 4
Cheing-03	TENS	30	3	2	50.3	32.2 (23.9 to 40.5)	**4**, 8
Defrin-05	IF	45	4	4	71.0	41.6 (33.4 to 49.8)	**4**
Fargas-Babjak-89	TENS	19	3	12	-	45.3 (11.7 to 78.9)	6, 12
Law-05	ALTENS/TENS	27	4	2	57.3	29.7 (7.6 to 51.3)	**2**, 4
Lewis-84**	TENS	29	3	3	-	7.0 (-5.6 to 19.6)	**3**
Lewis-94**	TENS	28	1	3	-	4.9 (-8.4 to 18.3)	**3**
Smith-83	TENS	15	3	4	-	dichotomous data only	**4**
Taylor-81**	TENS	10	2	2	-	5.5 (-7.3 to 17.8)	**2**
Yurtkuran-99	ALTENS	25	3	2	-	20.0 (14.1 to 25.9)	**2**
Best within 4 weeks, all trials		223				18.8 (9.6 to 28.1)	
Best within 4 weeks, optimal trials		156				22.2 (18.1 to 26.3)	
8 weeks		30				8.3 (-1.1 to 17.6)	
Global improvement 8 weeks		15				1.8 (0.6 to 4.8) Relative Risk	
Overall		259	3.3*		63.8†		2.8*

*Mean † Weighted mean – Not Available ** Trials with non-optimal treatment

**Table 3:** Characteristics of included trials of electro-acupuncture for pain relief in patients with knee osteoarthritis

**First author, publication year**	**Type**	**No of patients on active therapy (n = 121)**	**Method quality**	**Treatment period (weeks)**	**Mean baseline pain (mm VAS)**	**Best mean difference (95% CI) of change over placebo (mm VAS)**	**Outcome assessment timepoints (weeks, best time point used in bold)**
Sangdee-03	Maximal tolerable intensity	48	5	4	66.9	25.0 (15.2 to 34.8)	**4**, 8, 12
Vas-04	Maximal tolerable intensity	48	4	12	58.9	25.0 (13.4 to 36.6)	12
Yurtkuran-99	Maximal tolerable intensity	25	3	2	-	20.0 (14.1 to 25.9)	**2**
12 weeks Global improved		48				2.1 (1.1 to 4.1) Relative Risk	
Best within 4 weeks		73	4*		62.9†	21.3† (16.3 to 26.3)	3*
Total		121	4*		62.9†	21.9† (17.3 to 25.3)	3*

*Mean † Weighted mean

**Table 4:** Characteristics of included trials of manual acupuncture for pain relief in patients with knee osteoarthritis

**First author, publication year**	**Type**	**No of patients on active therapy (n = 409)**	**Method quality**	**Treat-ment period (weeks)**	**Mean baseline pain (mm VAS)**	**Best mean difference (95% CI) of change over placebo (mm VAS)**	**Outcome assessment timepoints (weeks, best time point used in bold)**
Berman-04	TCM with "de Qi"	169	4	12	44.5	1.2 (-2.5 to 4.9)	**4**, 8
Molsberger-94	TCM with "de Qi	71	4	5	46.8	10.6 (0.1 to 21.3)	5
Takeda-94	TCM with "de Qi"	20	3	4	56.0	2.0 (-7.5 to 11.9)	**4**
Witt-05	TCM with "de Qi"	149	4	8	64.9	7.1 (0.2 to 14.4)	8
8 weeks		318				3.6 (0.2 to 7.1)	
Total		409	3.8*		54.7†	1.3 † (-2.7 to 4.7)	4

*Mean † Weighted mean

**Table 5:** Characteristics of included trials of Low Level Laser Therapy for pain relief in patients with knee osteoarthritis

**First author, publication year**	**Intervention type, treatment period**	**No of patients on active therapy (n = 187)**	**Method quality**	**Mean baseline pain (mm VAS)**	**Best mean difference (95% CI) of change over placebo (mm VAS)**	**Outcome assessment timepoints (weeks, best time point used in bold)**
Bulow-94**	25 mW, 830 nm, 2.25 J to 10 points 3 times/week, 9 total sessions	15	2	82.0	8.0 (-10.8 to 26.8)	**3**, 6
Gøtte-95	12 mW, 904 nm, 12 J in 4 points, 3 times/week 12 total sessions	20	3	69.0	25.0 (9.4 to 40.7)	**4**
Gur-03	10 mW, 904 nm, 2 or 3 J applied to 2 points, 2–3 times week/10 total sessions	60	4	73.9	25.0 (18.9 to 31.1)	**4**, 8, 12
Hegedu-06	50 mW, 830 nm, 6 J applied to 8 points, 2 times/week, total 8 sessions	14	3	57.5	25.1 (2.5 to 47.7)	**4**, 6, 12
Nivbrant-92	4 mW 904 nm, 0.7 J in 3 points, 3 times/week, 6 total sessions	15	4	67	19 (2.4 to 35.6)	**2**, 6
Stelian-92	2.7 mW and 25 mW 904 + 820 nm, 1.3 J and 11.1 J in two points 10 times/week, 20 total sessions	18	4	72.0	41 (25.4 to 56.6)	**2**, 10
Tascioglu-05**	50 mW 830 nm, 1.5 or 3 J in 5 points, 5 times/week, 10 total sessions	20	3	66.5	-0.9 (-11.8 to 10)	**3**
Yurtkuran-06 **	4 mW, 904 nm, 0.48 J in one acupoint (Sp9), 5 times/week, 10 total sessions	25	5	-	-0.7 (-18.6 to 17.2)	**2**, 12
Best within 4 weeks, all trials††		187			17.7 † (8.1 to 27.3)	
Best within 4 weeks, optimal		142			24.2 (17.3 to 31.1)	
6–8 weeks		104			15.5 (9.9 to 20.9)	
12 weeks		99			12.3 (6.7 to 17.9)	
Total		187	3.5*	70.3†		3*

*Mean † Weighted mean †† Random effects model used for calculation ** Non-optimal dose

**Table 6:** Characteristics of included trials of pulsed electromagnetic fields for pain relief in patients with knee osteoarthritis

**First author, publication year**	**Intervention type, treatment sessions and period**	**No of patients on active therapy (n = 255)**	**Method quality**	**Mean baseline pain (mm VAS)**	**Best mean difference (95% CI) of change over placebo (mm VAS)**	**Outcome assessment timepoints (weeks, best time point in bold)**
Callaghan-05	SWT, 3 sessions/week, 6 sessions total, output 20 W in 20 minutes, 400 Hz, treatment dose 24 kJ	9	5	65.0	15.0 (-12.7 to 42.7)	**2**
Jacobson – 01	PEMF, 8 sessions, 1–8 Hz, 3 × 10^-7^G	101	3	63.3	7.9 (0.8 to 15.4)	**2**, 4
Nicolakis-02	PEMF 30 min daily, 6 weeks, 40 mT 1–3000 Hz	15	4	34.6	10.8 (-3.5 to 25.1)	6
Pipitone-01	PEMF 30 min daily, 6 weeks, 3–20 Hz, <0.5 Gauss	34	5	-	2.0 (-5.8 to 9.7)	6
Thamsborg-05	PEMF, 2 h daily for 6 weeks, 10 mV, 50 Hz	42	5	52.6	0.3 (-7.7 to 7.9)	**2**, 6, 12
Trock-93	PEMF, 15 Gauss, <30 Hz, 30 min 3–5 times/week, total 18 sessions	14	4	76.5	31.0 (11.0 to 51.0)	2,**4**, 8
Trock-94	PEMF, 15 Gauss, <30 Hz, 30 min 3–5 times/week, total 18 sessions	40	5	70.7	14.6 (0.9 to 28.3)	2, **4**, 8
Best within 4 weeks		209			6.9† (2.2 to 11.6)	
6 weeks		91			1.0 (-4.1 to 6.0)	
8 weeks		47			19.8 (7.1 to 32.5)	
12 weeks		42			-2.4 (-10.1 to 5.3)	
Pooled 6–12 weeks		180			4.8 (-2.2 to 11.8)	
Total		255	4.4*	63.7†		3.2*

*Mean † Weighted mean

**Table 7:** Characteristics of included trials of ultrasound therapy for pain relief in patients with knee osteoarthritis

**First author, publication year**	**Type**	**No of patients on active therapy (n = 74)**	**Method quality**	**Mean baseline pain (mm VAS)**	**Best mean difference (95% CI) of change over placebo (mm VAS)**	**Outcome assessment timepoints (weeks, best time point in bold)**
Falconer-92	Maximal tolerable 0.1 to 2.5 W/cm2. 2–3 sessions/week, 12 sessions total	74	4	53.0	n.s.	**4**
Total		74	4	53.0	n.s.	**4**

*Mean.

† Weighted mean

**Table 8:** Characteristics of included trials of static magnets for pain relief in patients with knee osteoarthritis

**First author, publication year**	**Type**	**No. of patients on active therapy (n = 86)**	**Method quality**	**Treatment period (weeks)**	**Mean baseline pain (mm VAS)**	**Best mean difference (95% CI) of change over placebo (mm VAS)**	**Outcome assessment timepoints (weeks, best time point used in bold)**
Hinman-02	Static magnets on knee"	22	4	2	38.8	8.6 (1.4 to 15.4)	**2**
Harlow-04	Static magnets on wrist (bracelet)	64	5	12	66.8	1.7 (-5.2 to 8.6)	**4**, 12
Best within 4 weeks		86				5.1 (0.2 to 10.0)	
12 weeks		64				6.5 (-0.6 to 13.6)	
Total		86	3.8*		54.7†		4

*Mean † Weighted mean

### Short-term efficacy, best effect within 4 weeks

Six out of the 36 included trials did not provide continuous data within 4 weeks from treatment start. Two of these trials were on TENS [40,41], one trial on EA [42] and one trial on MA [43].

**Figure 2:**
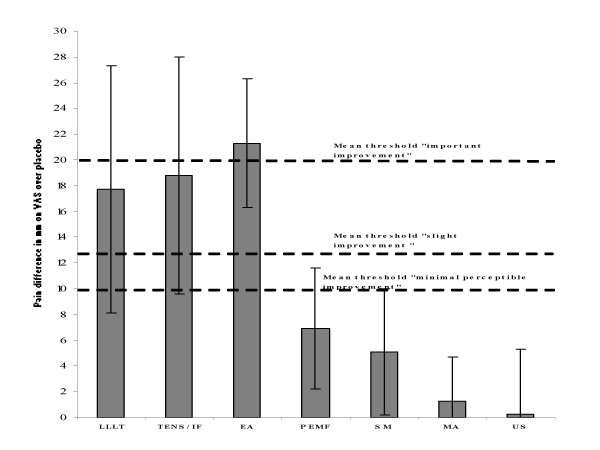
**Primary outcome**. Efficacy for each intervention measured at the end of treatment. Mean difference over placebo for pain measured on a 100 mm visual analogue scale (VAS) is shown as columns, and error bars indicate 95% confidence limits. The horizontal dotted lines indicate subjective thresholds for mean perceptible improvement (lowest), mean slight improvement (middle) and mean important improvement (top). Abbreviations: LLLT (Low Level Laser Therapy), TENS/IF (Transcutaneous Electrical Nerve Stimulation and Interferential Currents), EA (Electro-acupuncture), PEMF (Pulsed Electro Magnetic Fields), MA (Manual Acupuncture), US (Ultrasound).

The primary outcome, i.e. the overall best efficacy of the different physical interventions within 4 weeks, is summarised in Figure 2.

### Secondary outcome – pain relief at follow-up

For the secondary outcome, pain relief at follow-up 0 – 12 weeks after end of treatment, there was a paucity of data for manual acupuncture, TENS/IF and EA, which precluded a firm assessment of the efficacy for these interventions. However, when estimates of the continuous and categorical data of global improvement for TENS and IF was combined, data suggest that most of the pain relieving effect is retained for at least 2 weeks [44], or 4 weeks [40,45] after the end of treatment (see also table 2). For EA, the same tendency was seen, with global improvement lasting 8 weeks after the end of treatment [46] (see also table 3). For PEMF results were conflicting when comparing efficacy at different time-points. Data for LLLT indicated a slowly decreasing trend over time, which was still giving a slight pain relief up to 8 weeks after treatment was stopped [47,48] (see also table 5).

### Side-effects and adverse reactions

Six of the LLLT-trials [49,37,47,52] stated that treatment was safe and/or that no adverse effects were observed. One TENS trial reported 4 patient withdrawals (14%) for unspecified adverse effects from TENS treatment [39], and 1 TENS-trial reported mild skin reactions after treatment [53]. Four TENS trials stated no withdrawals due to adverse events [44,45,54,55], while 2 trials did not report on withdrawals or drop-outs. One withdrawal (2%) was reported in each of the two EA trials due to increased pain or unspecified cause [42,46], and no withdrawals were reported in the last EA trial [55]. For MA, 14% of the patients reported mild side effects such as small haematomas. None of the withdrawals in the 2 MA trials were related to the therapy given trial [56,57]. For PEMF, three trials [58-60] stated that no adverse events had occurred. In 1 PEMF trial one patient withdrew after reporting increased pain during treatment [61]. For SM, 2 patients (3%) reported dizziness or increased pain in 1 trial [62].

### Subgroup analysis of methodological quality

Trials were generally of medium to high quality (≥ 3), with the exception of one LLLT trial with method score 2 [37] and two TENS trials with method scores 1 [39] and 2 [36]. Exclusion of these trials from meta-analyses increased efficacy slightly for the two interventions in question to 23.3 mm (95% CI 13.4 to 33.1) and 18.5 mm (95%CI 8.5 to 29.2) for TENS and LLLT respectively.

### Subgroup analysis of trials with optimal doses

**Figure 3:**
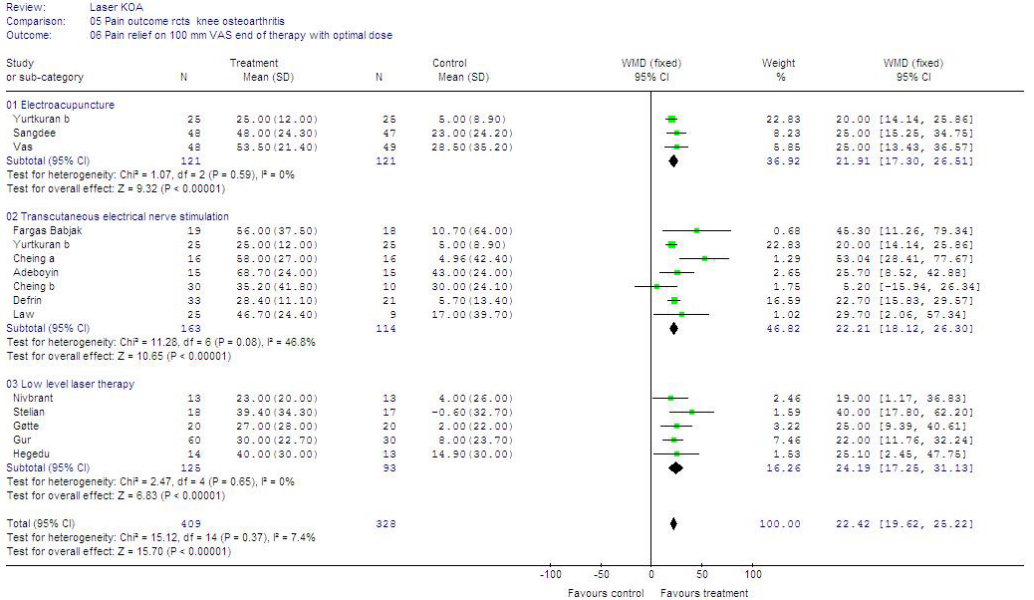
**Primary outcome – Forest plot for subgroups with optimal treatment**. Forest plot over 16 trials with optimal treament procedures and dose. Trials plotted on the right hand side of the middle line (WMD (fixed)) indicates a positive treatment effect. The combined effect size for each intervention is placed below the trials, and combined overall effect of all 16 trials is plotted on the bottom.

Seven trials had to be excluded from the pre-planned subgroup analysis with known optimal dose ranges for EA, LLLT and TENS. One EA trial did not administer strong, near-noxious electrical current intensity [57], and neither did three TENS crossover trials [36,39,63]. For LLLT, one trial with 904 nm delivering 0.48 J in one point, and one trial with 830 nm administering 3J in five points had too low doses and did not comply with WALT dosage recommendations. The results of the subgroup analyses showed that EA (21.3 mm [95%CI 16.3 to 26.3]), LLLT (24.2 mm [95% CI 17.3 to 31.1]) and TENS (22.2 mm [95% CI 18.1 to 26.3]) offered similar and clinically relevant relevant pain relief. The results are summarised in by trials in figure 3.

### Clinical relevance of effects related to patient-centered outcomes

**Figure 4:**
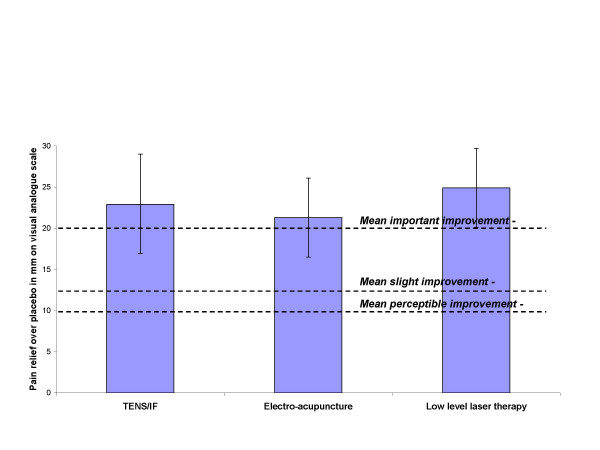
**Primary outcome – subgroups**. Best efficacy for optimal dose and administration of each intervention within 4 weeks after treatment start. Mean difference over placebo for pain measured on a 100 mm visual analogue scale (VAS) is shown as columns, and error bars indicate 95% confidence limits. The horizontal dotted lines indicate subjective thresholds for mean perceptible improvement (lowest), mean slight improvement (middle) and mean important improvement (top). Abbreviations: LLLT (Low Level Laser Therapy), TENS/IF (Transcutaneous Electrical Nerve Stimulation and Interferential Currents), EA (Electro-acupuncture).

For optimal treatment with EA, TENS and LLLT, the combined results indicated clinically relevant and important effects when related to patient-centered outcomes (Figure 4).

### Subgroup ananalysis of funding sources

Most of the trials were independently funded by unrestricted research grants from independent sources or the hospitals where the trials were taking place. None of the TENS/IF, LLLT, MA or EA trials were funded by for-profit organisations. Three of the PEMF-trials were funded by the supplier of the equipment [59-61], and excluding these industry-funded trials from analysis reduced efficacy to non-significance at 2.8 mm (95% CI – 3.7 to 9.2).

### Publication bias

**Figure 5:**
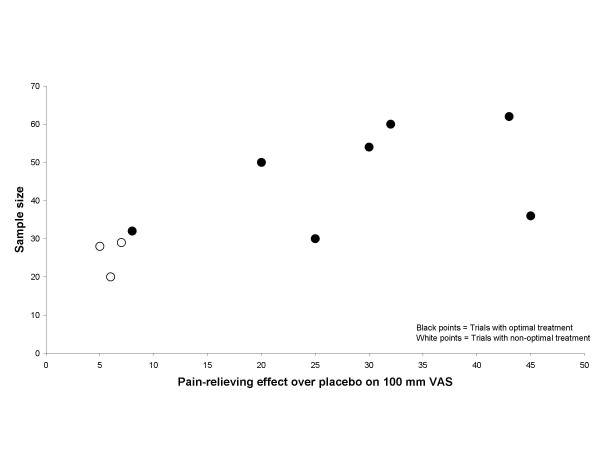
**Effect size plot TENS/IF trials**. Effect/size plot for TENS/IF trials. Open circles indicate trials with non-optimal treatment, and black circles indicate trials with optimal dose and treatment procedure. Effect over placebo is related to the x-axis and sample size is related to the y-axis.

**Figure 6:**
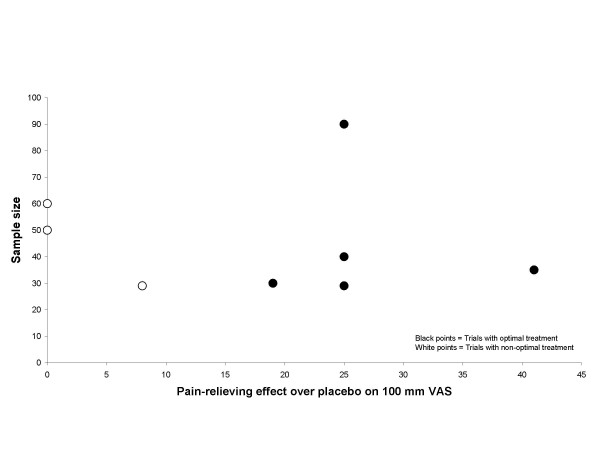
**Effect size plot LLLT trials**. Effect/size plot for LLLT trials. Open circles indicate trials with non-optimal treatment, and black circles indicate trials with optimal dose and treatment procedure. Effect over placebo is related to the x-axis and sample size is related to the y-axis.

**Figure 7:**
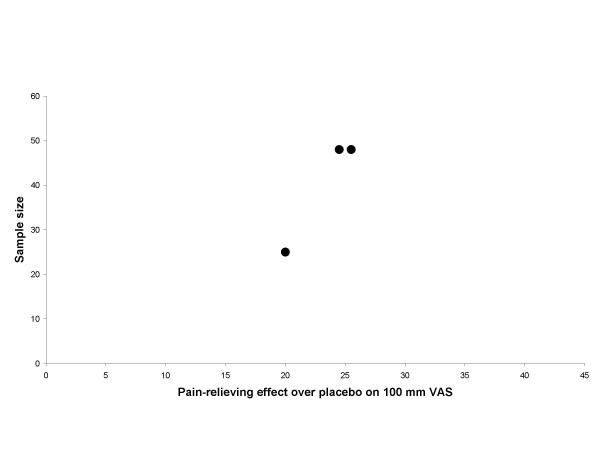
**Effect size plot EA trials**. Effect/size plot for EA trials. Black circles indicate trials with optimal dose and treatment procedure. Effect over placebo is related to the x-axis and sample size is related to the y-axis.

**Figure 8:**
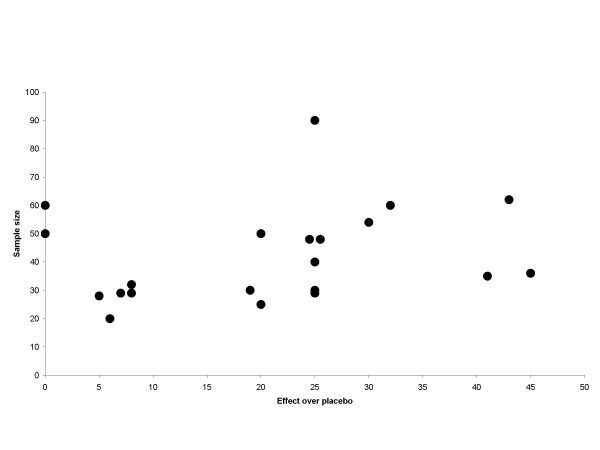
**Publication bias plot**. Effect/size plot for trials with TENS/IF, LLLT and EA. Black circles indicate a single trial. Effect over placebo is related to teh x-axis and sample size is related to the y-axis.

The graphical plots showed no obivious evidence for publication bias, but the number of included trials was small (Figure 5, 6, 7 and 8).

## Discussion

It seems that all but two of the included physical agents (MA and ultrasound therapy), exhibit statistically significant effects over placebo within 1–4 weeks, regardless of what doses and treatment procedures were being used. However, effect sizes for PEMF and SM, failed to reach the mean threshold for "minimal perceptible clinical improvement" for OAK as defined by Ehrich et al. [16]. It cannot be ruled completely out that more studies may contribute to optimise PEMF treatment procedure and dosage, but at present MA, PEMF, US and SM cannot be recommended for rapid pain relief in OAK management.

For TENS, the above findings are at odds with previous reviews of TENS in chronic pain [64] and in chronic low back pain [65], but not in knee osteoarthritis [66].

The picture for acupuncture is mixed, and most studies with MA have been performed using fewer weekly treatment sessions than the other interventions. Consequently, the results at 4 weeks are similar to those of the placebo groups, while the effect at 8 weeks is statistically superior to placebo. In a systematic review of acupuncture reviews, it has been argued that the evidence in favour of acupuncture is weakened by lack of randomisation and lack of assessor or patient blinding [67]. In this review, we have only included randomised and double-blinded (patient and assessor) trials, and the results are in line with a recent review of acupuncture OAK [68]. However, the clinical relevance of the MA effect in OAK remains questionable, and the results infer that EA seems to be a better choice in OAK management.

For LLLT, a Cochrane review has found limited evidence in favour of LLLT in rheumatoid arthritis and inconclusive evidence in osteoarthritis [69]. But we have previously pointed out that the findings in osteoarthritis could be caused by inherent methodological weaknesses [70] such as lack of adequate dose-response analyses[71]. In line with the dosage recommendations from World Association for Laser Therapy, the findings above suggest that 904 nm is only effective with doses of 2–12 Joules and 830 nm with doses of 20–48 Joules when applied to 2–8 points over the joint capsule.

The small sample size of some trials on EA, TENS and LLLT may undermine the validity of our conclusions. It has been argued that evidence for most interventions lack sufficient statistical power to make valid conclusions [72]. The Oxford pain research group suggests that reasonably robust conclusions can be be made from systematic reviews including 200 patients and/or more than 4 trials [67]. Cochrane reviews offer positive conclusions for pharmacological interventions for pain based on the inclusion of 40 patients for neck pain [73] and 185 patients for OAK [74]. The sample size for our total and subgroup analyses for optimal treatment for EA, TENS and LLLT met the criteria stated by the Oxford group (EA n = 242, LLLT n = 222, TENS n = 272). Nevertheless, we remain cautious in our conclusion until larger scale clinical trials are available to verify the results. Methodological trial quality also undermines review conclusions [36,63], although the majority of trials on which our conclusions rest, were of acceptable quality.

The biological rationales for the observed effects seem somewhat clearer for EA, TENS and LLLT than for the interventions that demonstrated lesser effects. EA and TENS has been shown to inhibit ongoing nocicpetive transmission at a segmental level and that this is dose-dependent [75]. The EA-trials included in the review delivered electrical stimulation with needles placed in the painful area, similar to that used for TENS. This is consistent with established physiological principles whereby stimulation in dermatomes and myotomes related to the pain are likely to elicit segmental analgesia mechanisms. It has been shown in experimental studies that electrical stimulation by both needle and skin electrode can produce similar analgesic effects [76]. The observed similarities between TENS and EA in effect size and time-effect profiles after cessation of treatment, may be indices that similar physiological mechanisms are being induced by these two interventions. Adding the data from trials using acupuncture to trials using electrical stimulation in the form of EA, did not increase effect size over TENS to any appreciable extent.

During the last three years, controlled LLLT-trials have found dose-dependent anti-inflammatory effects under *in vitro*, *in vivo*, and *in situ *conditions [77,78]. Another possible explanation for the observed positive LLLT effects may arise from local dose-dependent biostimulatory effects on cell activity which have been observed in controlled *in vitro *[79]*and vivo *[80] trials with lower, but overlapping, dose intervals.

The value of standardising treatment procedures and dosage in the treatment with physical agents is highlighted by the finding that doses which work well in laboratory settings also can be extrapolated to induce better pain reduction in the clinical subgroups of EA, TENS and LLLT-trials with optimal treatment. But the heterogeneity of treatment procedures, application techniques and doses still call for careful interpretation of the results.

Until now, physical therapies have often been neglected in editorials and reviews of treatments for OAK [81,82] and this may have resulted in the under-utilization of physical agents in OAK management [83]. The safety of the physical therapies seems good as no serious adverse events were reported in the 36 RCTs reviewed. The advantage of physical agents is that they can be used in combination with drug therapy, thus reducing drug dosage and adverse effects. There is also some evidence that effects from adequately administered TENS, EA and LLLT remain clinically relevant even 1–2 months after the end of treatment. It may be difficult to directly compare the results of trials of physical agents with those of pharmacological interventions because of differences in the nature of the placebo's used in the trials.

In the pharmacological literature publication bias in favour of small trials with positive results has previously been detected. There seems to be no support for this tendency from asymmetry in the graphical plot [27]. On the contrary, a small asymmetry towards publication bias in favour of small trials with negative results seems to be present for these physical interventions.

Exercise therapy, education and weight loss still remain the cornerstones of long-term OAK management [81], but our results suggest that EA, TENS and LLLT have potential to become useful adjuncts in OAK pain management.

## Conclusion

For patients with x-ray grade 2–4 and pain intensity levels above 50 mm on VAS, an intensive regimen of 2–4 weeks with TENS, EA or LLLT seems to safely induce statistically significant and clinically relevant short-term pain relief.

## Competing interests

The author(s) declare that they have no competing interests.

## Authors' contributions

JMB and AEL conceived of the study, and participated in its design and coordination BB, and RLM performed the literature search in the databases, while MIJ and RC handsearched for additional studies. JMB, BB and AEL and RLM made the methodological assessment of studies, and JMB, RLM and RC performed the statistical analysis and the meta-analyses. JMB, MIJ and RC drafted the manuscript, BB made the figures and AEL revised the manus for typographical errors. All authors read and approved the final manuscript.

## Pre-publication history

The pre-publication history for this paper can be accessed here:


